# Influence of silver nanoparticle solution on the mechanical properties of resin cements and intrarradicular dentin

**DOI:** 10.1371/journal.pone.0217750

**Published:** 2019-06-26

**Authors:** Thaís Yumi Umeda Suzuki, Juno Gallego, Wirley Gonçalves Assunção, André Luiz Fraga Briso, Paulo Henrique dos Santos

**Affiliations:** 1 Department of Restorative Dentistry, Faculty of Dentistry, Federal University of Minas Gerais—UFMG, Belo Horizonte, MG, Brazil; 2 Department of Mechanical Engineering, Engineering Faculty of Ilha Solteira, São Paulo State University, Ilha Solteira, SP, Brazil; 3 Department of Dental Materials and Prosthodontics, Aracatuba School of Dentistry, São Paulo State University, Araçatuba, SP, Brazil; 4 Department of Restorative Dentistry, Aracatuba School of Dentistry, São Paulo State University, Araçatuba, SP, Brazil; National Taiwan University, school of dentistry, TAIWAN

## Abstract

This study evaluated the influence of silver nanoparticle on mechanical properties of the components of underlying dentin and resin cement in different regions of intraradicular dentin. Ninety extracted single-rooted human teeth were used in this study. After endodontic preparation, the teeth were divided into five groups, according to the irrigating agents: distilled water, 5.25% sodium hypochlorite, 25% polyacrylic acid, 2% chlorhexidine and 23 ppm silver nanoparticles dispersion. Then, the groups were divided in 3 subgroups (n = 6) according to the technique adopted for adhesive cementation: SUA group: Scotchbond Universal Adhesive + RelyX ARC; U200 group: RelyX U200; and MCE group: MaxCem Elite. The mechanical properties of hardness and elastic modulus were measured in resin cement and underlying dentin in ultra-micro hardness tester in different thirds of radicular dentin surface. Data were subjected to ANOVA and Fisher’s test (p = 0.05). In the underlying dentin, in general, there was no statistically significant difference in different thirds of intraradicular dentin according to the different solutions used. In the resin cements, higher hardness values were found, in general, for the cervical third. When silver nanoparticle solution was used, higher mechanical properties were generally obtained for resin cement for the SBU and U200 groups, with little or no changes in mechanical properties for the dentin. Silver nanoparticle application is a viable option for irrigation the intraradicular dentin previously through the cementation process of glass fiber posts. The mechanical properties are influenced by irrigant solutions used and the depth intraradical analyzed area.

## Introduction

Restoring endodontically treated teeth with glass fiber posts when the remaining tooth structure cannot provide adequate support and retention for the restoration has become popular [1,2]. During the post space preparation, a debris and smear layer is formed on the canal walls, leading to increased leakage and obstruction of the dentin tubules, thus blocking the adhesive luting of the fiber post [1]. Moreover, the removal of the root canal filling while preparing the post space without rubber dam isolation or inadequate temporary restorations may cause the invasion of microorganisms through oral fluids into the canal, which could result in failure of the bonding process [1].

In this case, the use of conditioning agents seems be important to ensure bacterial minimization and elimination of organic tissue remnants [3] because some studies reported a low bond strength of self-adhesive resin cements to intraradicular dentin [2,4,5]. Several products are currently used as endodontic irrigants, such as distilled water, sodium hypochlorite (NaoCl) [1,6,7], chlorhexidine gluconate [1,8] and polyacrylic acid [9].

Recently, silver nanoparticles (Ag NPs) have gained popularity in health sciences as an antimicrobial and anti-inflammatory agent [3,10,11]. Silver is a metal known for its broad-spectrum bactericidal and virucidal properties [3,7,11]. The size of silver nanoparticle is an important characteristic because smaller particles reduce the particle concentration necessary for efficacy, since it give rise to higher specific surface areas [12]. It has been incorporated to many materials in Medicine in different forms [12–14], for example in nanofiber mats, bandages, wound dressings and ointments [3]. In Dentistry, Ag NPs have been demonstrated to be effective antimicrobial components in prosthetic materials, adhesives and implants, to promote caries arrestment, to prevent biofilm formation, and for osteogenic induction [15–17].Optical glass fibers coated with Ag NPs have also been proposed to use for endodontic therapy as a system of root dental fillings. This is due to their antibacterial and mechanical properties on the glass fiber cores surface. [18]

However, Ag NPs have not yet been evaluated for use in post space preparation especially as an irrigant solution. The purpose of this study was to evaluate the mechanical properties (hardness and elastic modulus) of the components of the adhesive interface (underlying dentin and resin cement) in different regions of intraradicular dentin (cervical, middle and apical) submitted to different conditioning agents (distilled water, 5.25% sodium hypochlorite, 25% polyacrylic acid, 2% chlorhexidine and 23 ppm silver nanoparticle dispersion). Two null hypotheses were tested: that the interaction between the conditioning agents and bonding materials would not cause changes in the mechanical properties (Martens hardness [MH] and elastic modulus [Eit*]) of the resin cement and underlying dentin and that no difference would be found in the mechanical properties of the components of the adhesive interface in different thirds of intraradicular dentin.

## Materials and methods

The materials used in this study are listed in Table 1. Ninety single-rooted human premolars from different individuals, extracted for orthodontic or periodontal reasons, were used in this study. The use of extracted human teeth was performed in accordance with the guidelines of the ethics committee of the Sao Paulo State University and all patients signed informed consent forms before enrollment. The protocol for this study was approved Research and Ethics Committee of the Araçatuba School of Dentistry, Sao Paulo State University (Protocol #05142812.4.0000.5420). All teeth exhibiting clinical signs of caries, root resorption, cracks, or fractures were excluded.

**Table 1:** Materials used in study.

Trademark	Product	Composition*	Manufacturer
Scotchbond Universal Adhesive	Total-etch, selective etch, self-etch adhesive	MDP phosphate monomer, dimethacrylate resins, HEMA, Vitrebond copolymer, filler, ethanol, water, initiators, silane.	3M ESPE, St. Paul, MN, USA
Scotchbond Universal DCA	Dual Cure Activator	Sodium toluene sulfinate, ethanol.	3M ESPE, St. Paul, MN, USA
RelyX ARC	Conventional resin cement	Base paste: Bis GMA,TEGDMA, benzoyl peroxide. Catalyst paste: Bis GMA, TEGDMA, photoinitiator system, amine, peroxide, zirconia/sílica filler 67.5% by weight	3M ESPE, St. Paul, MN, USA
RelyX U200	Self-adhesive resin cement	Base paste: Methacrylate monomers containing phosphoric acid groups, methacrylate monomers, silanated fillers, initiator components, stabilizers, rheological additives. Catalyst paste: methacrylate monomers, alkaline (basic) fillers, silanated fillers, initiator components, stabilizers, pigments, rheological additives.	3M ESPE, St. Paul, MN, USA
Maxcem Elite	Self-adhesive resin cement	Uncured methacrylate ester monomers, non-hazardous inert mineral fillers, ytterbium fluoride, activators, stabilizers, colorants.	Kerr, Orange, CA, USA

*According to the manufacturers.

### Endodontic treatment

The anatomic crowns of all teeth were removed 1.0 mm above the cementum-enamel junction through a transversal section under water cooling with a low-speed diamond saw (Isomet 2000; Buehler, Lake Bluff, IL, USA). The specimens were then endodontically treated. A #10 K-file (Maillefer Instruments) was introduced into the root canal until it was visible at the apical foramen. The working length was determined to be 1.0 mm less than this length. The root canals were instrumented with K-files with size of up to #45 by using the crown-down and step-back techniques after the preparation process had been completed. This process was performed with Hedstroem files of up to #60 scaled to be natural and progressive and used as an instrument of memory a K-file 2 number lower than the larger caliber used in the apical preparation. Throughout the preparation process, the root canals were irrigated with 2.5% sodium hypochlorite. The instrumented root canals were dried with sterile paper points and immediately obdurated by the lateral condensation of gutta-percha cones (Dentsply-Maillefer, Ballaigues, Switzerland) and Sealapex calcium hydroxide cement (Kerr, Orange, CA, EUA). Coronal access was sealed with Coltosol zinc oxide/zinc sulfate cement (Vigodent, Rio de Janeiro, RJ, Brazil). The endodontically treated teeth were stored in water at 37°C for 7 days.

### Preparing the post space for the cementation

The glass-fiber post system used in this study was Reforpost #3 (Ângelus, Londrina, PR, Brazil). The post spaces in all the specimens were prepared with a #2, 3, 4 and 5 low-speed drill (Dentsply-Maillefer) sequentially. The gutta-percha cones were removed to a depth of ±9 mm with reference to the working length of the tooth. After the completion of the preparation process, the adaptation of the fiber posts was verified to be complete by placing them in the post space. If the posts penetrated to the 9 mm depth, they were considered to have adapted.

### Fiber post surface treatment

Before the start of the adhesive procedure, the surfaces of the glass-fiber posts were treated with 35% phosphoric acid (3M ESPE, St. Paul, MN, EUA) for 60 seconds. They were then washed and air dried. The post surfaces were silanized for 60 seconds (Ângelus) and gently dried with an air jet. Finally, on the basis of the treatment to be performed in the intraradicular dentin, the adhesive system used on the dentin surface was also applied on the post surface if required. Thereafter, the post was not manipulated further to prevent contamination.

### Dentin treatment and fiber post cementation

The post spaces were irrigated with 2.0 mL distilled water to remove any gutta percha debris and to maintain the humidity of the environment. The root canal was dried with air and sterile paper points before the bonding procedures. The specimens were divided by drawing lots into the following 5 groups (n = 18) on the basis of the irrigating solutions: distilled water, 5.25% sodium hypochlorite (Apothicario, Araçatuba, SP, Brazil) [6], 25% polyacrylic acid (SDI, Bayswater, Austrália) [9], 2% chlorhexidine (Apothicario) [8] and 23 ppm silver nanoparticle dispersion (90μm Ag NP) (Khemia, São Paulo, SP, Brazil) [3]. The irrigation protocol of preparation for post cementation uses 5ml of each solution for a period of 60 seconds.

After irrigation, the specimens were divided by drawing lots into the following 3 subgroups (n = 6), according to the luting procedure used.

#### SUA group

The post spaces were dried with sterile paper points and the Scotchbond Universal Adhesive (3M ESPE) was applied along with Scotchbond Universal DCA. One drop of each solution was mixed for 5 seconds and applied actively inside of the preparation with a microbrush for 20 seconds, and then the adhesive was gently air dried for approximately 5 seconds to evaporate the solvent and light polymerized with a VALO Cordless (Ultradent, UT, EUA) for 10 seconds. The RelyX ARC conventional resin cement (3M ESPE) was mixed for 10 seconds and placed in the root canal with a #15 endodontic file. The resin cement was applied to the post surface and brought into position within the post space; any excess cement was removed. The resin cement was light polymerized for 40 seconds.

#### U200 group

The root canals were dried with sterile paper points and RelyX U200 self-adhesive resin cement (3M ESPE) was manipulated and inserted in the post space with a #15 endodontic file. The cement was applied to the post surface, the post was placed in position, and any excess cement was removed. Finally, the resin cement was light polymerized for 40 seconds.

#### MCE group

The root canals were dried with sterile paper points and Maxcem Elite (Kerr) was applied directly in the post space with the aid of a mixing tip. The cement was applied to the post surface, the post was paced in position, mild vibratory movements were made to avoid the possibility of air trapping, and any excess cement was removed. Finally, the resin cement was light polymerized for 40 seconds.

After the bonding process of the posts, all teeth had the coronal portion sealed with Z350 XT composite resin (3M ESPE); then, all teeth were stored for 7 days [19,20].

The teeth were submitted to thermal cycling (12,000 cycles; 5 to 55°C; dwell time: 30 seconds, transfer time: 2 seconds) using the thermal cycle simulation machine MSCT- 3 Plus (Marcelo Nucci, São Carlos, SP, Brazil) [21].

### Mechanical properties analysis

After cycling, the teeth were sectioned perpendicular to the long axis with a low-speed diamond saw under water cooling with an Isomet 2000 (Buehler) to obtain 1 slices approximately 1.0 mm in thickness of each third being analyzed (cervical, middle, and apical). The slices were embedded in acrylic resin (Classico, São Paulo, SP, Brazil); manually finished with #320, 600, 800 and 1200 grit silicon carbide paper (Extec Corp., Enfield, CT, EUA); and polished with diamond pastes (6, 3, and 1 μm) for a period of 4 minutes each. The specimens were cleaned in an ultrasonic unit (Model 2210; Branson Ultrasonic Corp., Danbury CT, EUA) with deionized water for 2 minutes between the steps and at the end of the process.

The Martens hardness (HM) and elastic modulus (Eit*) values were measured with an ultramicrohardness tester (DUH-211; Shimadzu, Kyoto, Japan) under a load of 5 mN at a speed of 1.5 mN/s; the holding time was 1 second. The regions analyzed were those corresponding to the components of the adhesive interface: resin cement, and dentin underlying the bonded interface. A Vickers tip was used, and 3 indentations were made in each region. The MH and Eit* values were calculated automatically by the software program installed with the tester. Statistical analysis were performed with 3-way analysis of variance (ANOVA), while considering the irrigating solutions, the luting procedures and the different regions of intraradicular dentin (cervical, middle and apical dentin) as the factors. Subsequently, Fisher’s protected least significant difference (PLSD) test (α = 0.05) was also performed.

## Results

### Dentin

**Fig 1:**
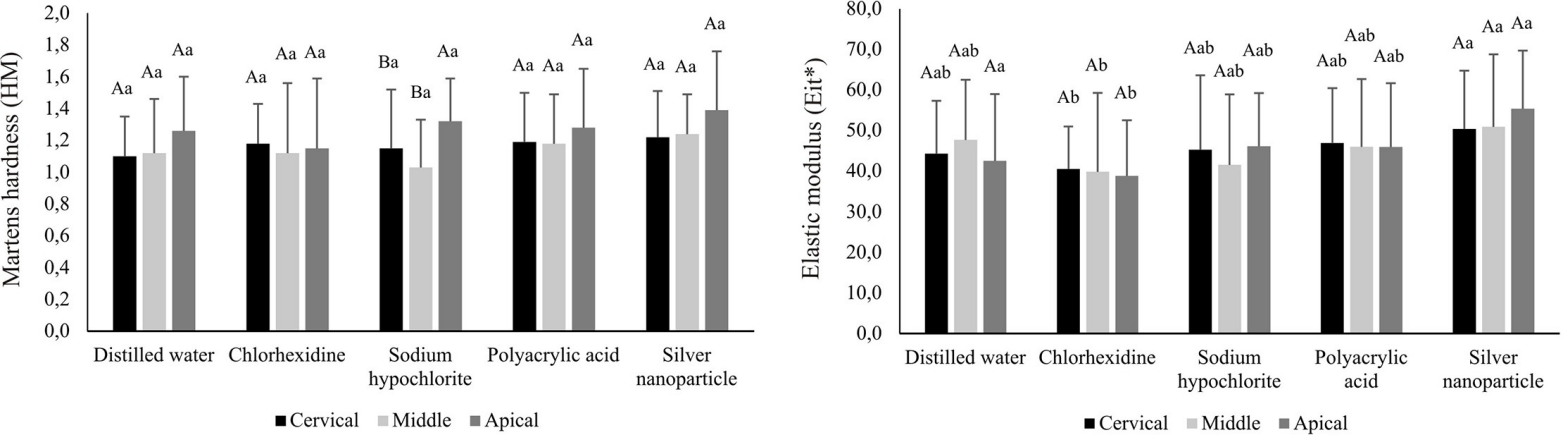
Mean (standard deviation) values of Martens hardness (HM) and elastic modulus (Eit*) of dentin categorized according to irrigating solutions and root third (GPa) independent of the material used for cementation**.

Fig 1 shows HM and Eit* values in different thirds of intraradicular dentin, according to the different irrigating solutions used. There was no significant difference between different regions in all experimental conditions for both properties analyzed (p>0.05) except for dentin irrigated with sodium hypochlorite, where the apical third showed higher HM values than cervical and middle thirds (p <0.05), regardless of the material used for cementation. The effect of the application of different irrigating solutions was demonstrated only in Eit* analysis, where irrigation with chlorhexidine has provided decrease in Eit* values compared to the silver nanoparticle solution in all regions studied (p <0.05).

### Resin cement

**Fig 2:**
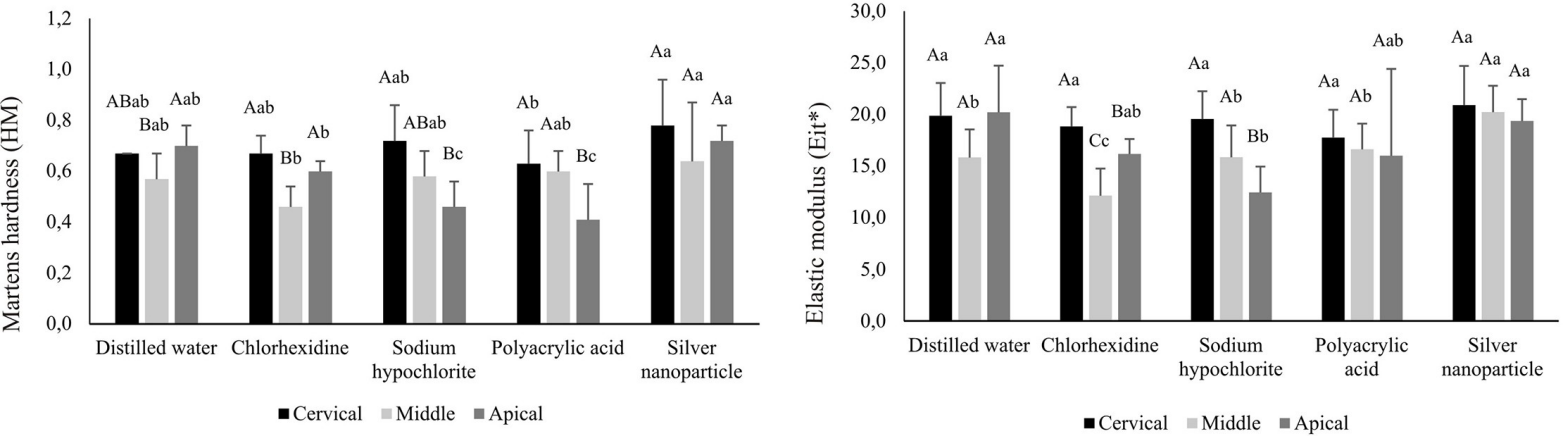
Mean (standard deviation) values of Martens hardness (HM) and elastic modulus (Eit*) of resin cement categorized according to irrigating solutions and root third (GPa) for SUA group**.

Fig 2 (SUA group), 3 (U200 group) and 4 (MCE group) show HM and Eit* of resin cements, in different thirds of intraradicular dentin. For the SUA group, there was decrease of HM values in the apical third when irrigated with sodium hypochlorite and polyacrylic acid compared to the cervical third (p<0.05). The same phenomenon occurred in the Eit* analysis, when irrigated with chlorhexidine or sodium hypochlorite (p<0.05). Comparing the solutions, independent the third analyzed, the highest values were found when the silver nanoparticle solutions was used (Fig 2), except for the apical third with distilled water.

**Fig 3:**
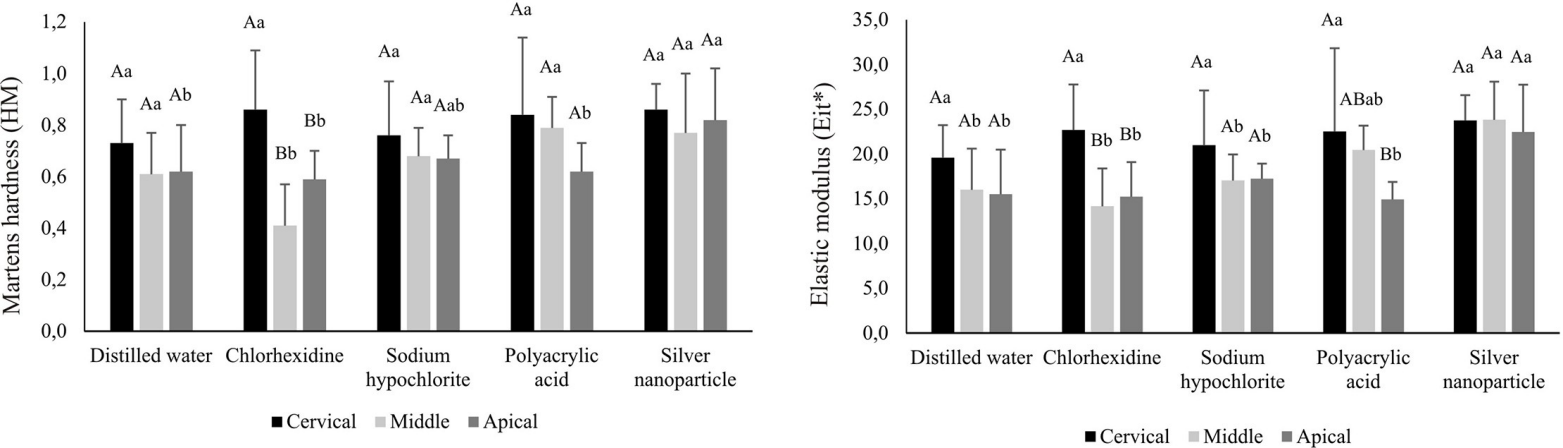
Mean (standard deviation) values of Martens hardness (HM) and elastic modulus (Eit*) of resin cement categorized according to irrigating solutions and root third (GPa) for U200 group**.

For the U200 group, the prior application of chlorhexidine causes a decrease in the mechanical properties of HM and Eit* in the middle and apical thirds, compared to cervical third (p<0.05). In the cervical third, there was no difference in both mechanical properties after irrigation with different solutions (p>0.05). However, in the middle and apical thirds, in general, the mechanical properties of the resin cement after application of the silver nanoparticle solution showed higher values compared to the other solutions (Fig 3).

**Fig 4:**
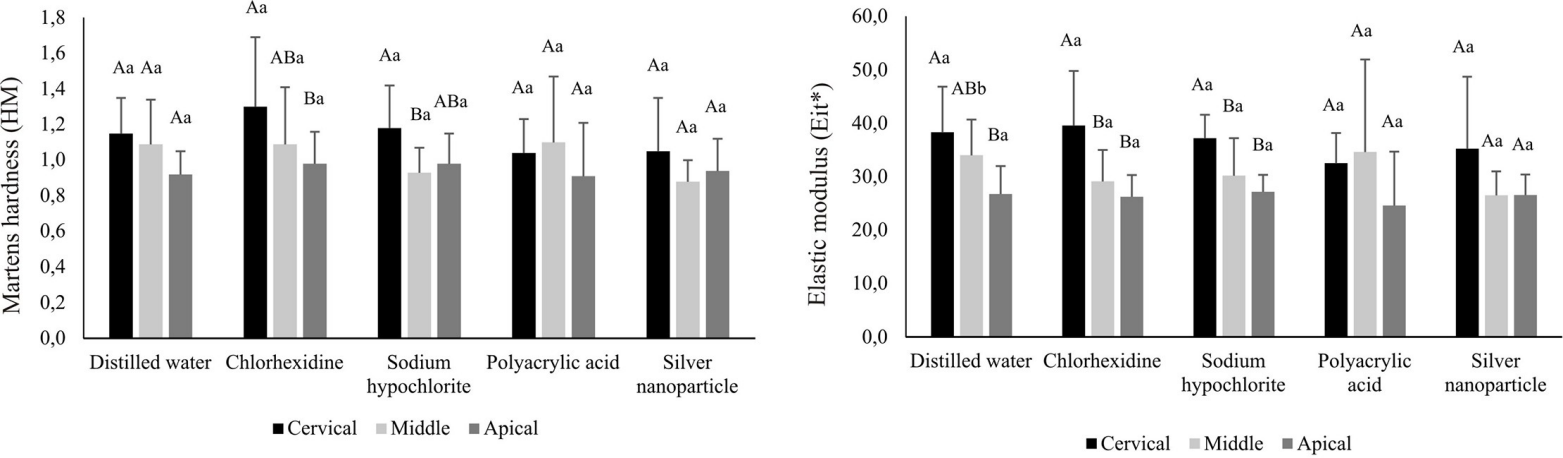
Mean (standard deviation) values of Martens hardness (HM) and elastic modulus (Eit*) of resin cement categorized according to irrigating solutions and root third (GPa) for MCE group**. ** Different uppercase letters indicate statistically significant differences among thirds and different lowercase letters indicate statistically significant differences among solutions (p < .05).

For the MCE group, in general, there was a decrease in mechanical properties of the resin cement in the cervico-apical direction, especially for Eit* (Fig 4), except for the groups that used polyacrylic acid and silver nanoparticles (p>0.05). There was no interference of different irrigating solutions on the mechanical properties of this material, compared to the control group for all regions studied (p>0.05).

## Discussion

The hardness and elastic modulus are mechanical properties that can be used to indirectly evaluate the degree of conversion of the material and, consequently, its polymerization effectiveness [22–25]. The present study evaluated the HM, whose calculation uses both plastic and elastic deformation, and the regional elastic modulus (cervical, medium and apical thirds) of the following structures: resin cement and underlying dentin to bonding interface. In general, the results found that the mechanical properties of the adhesive-interface components depend on the irrigating solutions, rejecting the first null hypothesis of this study. Furthermore, the mechanical properties varied during canal preparation, presenting differences between the thirds analyzed, which led to a rejection of the second null hypothesis.

The solution application with antimicrobial and/or conditioning activity would be clinically interesting, especially in cases where the cementation of intraradicular posts cannot be performed in the same clinical session of endodontic treatment. However, it is important that this solution was not able to change the mechanical properties of dentin because these solutions could not influence the behavior of the dentin-restoration interface [26,27], and reduce the fracture resistance of the root [26,28]. As the effect of the application of these solutions on dentin independent of cementing agent selected, the analysis of the properties of dentin were performed generalized, independent on the resinous material used (Fig 1). It was observed that the effect of the solutions occurred independently of the regions analyzed, except for the sodium hypochlorite solution. Although solutions have not caused statistically significant changes in HM, the dentin irrigated with the silver nanoparticle solution showed higher values compared to all solutions (Fig 1). The silver nanoparticle is a metal known for antibacterial activity of broad spectrum against gram-positive and gram-negative bacteria, fungi, protozoa and certain viruses [29]. It has been used to prevent bacterial colonization on different surfaces, such as catheters, prosthetics and clothes [30], and it can be used to reduce infection in burns [31] and water treatment [32].

The chlorhexidine although it has also been proven to have an antibacterial effect [33,34], decreased Eit* dentine compared to silver nanoparticles, when used in intraradical preparation (Fig 1) [26,35–39]. Chlorhexidine is a cationic compound with the ability to bind anionic molecules, such as phosphate, present in hydroxyapatite. Whereas phosphate is present in the calcium carbonate complex of dentin, chlorhexidine could induce changes in Ca-P relationship, which could explain the lowest values of the mechanical properties of dentin treated with this solution [21]. Based on these findings, it can be suggested that the silver nanoparticle solution would be a good alternative for the irrigation of preparation because it confirms the presence of bacterial activity and was not able to change the mechanical properties of dentin [3].

The application of sodium hypochlorite and polyacrylic acid did not cause changes in the HM and Eit* values of dentin compared to the control group (Fig 1). Sodium hypochlorite, in addition to its excellent antimicrobial potential, has the ability to dissolve the organic tissue of the dentin [7,26] into collagen and magnesium and phosphate ions [31]. Polyacrylic acid, in turn, would be able to promote cleaning the surface and increase the wetting capacity of the substrate [9,40] This acid is capable to remove partially the smear layer [9,41], leaving the mineral phase of the dentin and thus increasing the chemical reaction between the material and the substrate [42]. However, in this study, these changes do not reflect the possible change of the mechanical properties of the dentin. As the analysis of the mechanical properties was evaluated in the underlying dentin to the bonding interface, not specifically on the conditioned dentin, future studies should elucidate the effect of these agents in intraradicular dentin tissue.

Comparing the different thirds of intraradicular dentin, the apical third showed, in general, higher HM values. However, the difference was statistically significant only in the group irrigated with sodium hypochlorite (Fig 1). Apical sclerosis [19], cavity configuration factors [20,43], and difficulty viewing and accessing the apical part [36] could interfere proper contact of the solutions in the apical third. Thus, the cervical third would be more susceptible to contact with the irrigating solutions most likely to demineralization in this region [44,45]. However, these possible changes were not very evident in this study.

In post space preparation it is important that the antibacterial or dentin conditioning action solutions do not change the mechanical properties of the dentin and do not interfere in the polymerization process of cementing agents used. Thus, this study also assessed the effect of these agents in the irrigation mechanical properties of three resinous luting agents used in the fixing of intracanal retainers. In general, for the SUA and U200 groups, previous irrigation canal with the silver nanoparticle solution presented the highest values of HM and Eit*. (Figs 2, 3 and 4).

There are no studies evaluating the degree of conversion of resin materials when used in association with the silver nanoparticles solution. According to Ahn et al. (2009) [13], incorporation of silver nanoparticle into orthodontic adhesive did not affect the bond strength, which could indicate that there was no interference in the degree of conversion of material. Thus, it can be suggested that the degree of conversion of materials used in this study was not influenced by the silver nanoparticle solution, and it would therefore be a good option to use this solution before glass fiber post cementation.

In the SBU group, there was a decrease in HM and Eit* values in the apical third when previously irrigated with sodium hypochlorite, compared to the cervical third (Fig 2). Sodium hypochlorite is able to remove the organic components of dentine, including collagen, and it increases the penetration of the monomers in the demineralized dentin structure and dentinal tubules. However, after its application to the dentin, sodium hypochlorite decomposes into sodium chloride and oxygen. The oxygen usually causes the strong inhibition of polymerization of the adhesive material in the adhesive interface [46–48]. The formation of oxygen bubbles in the material-dentin interface can also interfere in the infiltration of the adhesive inside the tubules and demineralized dentin (Ari et al., 2003). Moreover, in the deepest thirds, sodium hypochlorite would not have been able to remove the smear layer, which could act as a barrier to dentin, reducing the permeability of dentin to bonding agents [48].

The previous irrigation of the root canal with chlorhexidine caused a decrease in the mechanical properties of RelyX U200 resin cement in the middle and apical thirds (Fig 3). The same phenomenon occurred in Eit* analysis of SUA group (Fig 2). The use of chlorhexidine might form precipitates resulting from the reaction between the phosphate present in dentin and the solution. These precipitates form a physical barrier, reducing the interaction between the luting material and the dentin surface [49]. In the deepest thirds, the accumulation of these precipitates would be higher because of the preparation’s configuration and the difficulty of viewing and access in this region [44]. As these groups are composed of self-etching and self-adhesive materials, the beneficial effect of chlorhexidine in minimizing the degradation of collagen under the action of MMPs [50] would be slightly evidenced.

For the MCE group, there was no interference of different irrigating solutions on the mechanical properties of this material compared to the control group for all studied thirds (Fig 4). There were, in general, decrease in the mechanical properties of the cement in a cervico-apical direction, with the exception for the groups which was performed before the application of polyacrylic acid and silver nanoparticles (Fig 4). The fact that the resin cements present higher properties in the cervical third were also found in other situations (Figs 2 and 3). Although all materials are classified as dual-curing materials, the proximity of the radiation source is a determining factor in the extension of the polymerization [51] because the light is not able to reach the deeper regions of the post space. Consequently, the resin material might not be completely polymerized in some regions. Thus, in these more apical regions, polymerization does not occur homogeneously. In the specific case of the SBU group, which used the Scotchbond Universal Adhesive with the Scotchbond Universal DCA and RelyX ARC dual-cured resin cement, according to the manufacturer, the dual cure activation would allow the adhesive system to be compatible with the dual or chemical resin cements enabling their polymerization, but does not change the adhesive that is solely photoactivated on a dual adhesive. Because the adhesive cannot be polymerized properly in the deepest thirds, residual acid monomers that are present in the adhesive layer react with the tertiary amine in the resin cement, which has an alkaline pH. Thus, the amine would be neutralized and the benzoyl peroxide in cement is not reduced, a reaction that is responsible for the polymerization of composite [52,53]. Moreover, the use of translucent posts could transmit the light more efficiently toward the middle and apical thirds, promoting polymerization because these posts could absorb, reflect and disperse light through the root canal.

The MCE group presented, in general, higher mechanical properties of HM and Eit* compared to other materials used, rejecting the third null hypothesis of the study. Previous studies [54,55] demonstrated that the Maxcem Elite self-adhesive resin cement showed higher degree of conversion values when compared with the RelyX Unicem or RelyX U200 resin cements, regardless of activation mode, photoactivation or just chemical activation. This could explain the higher hardness values for Maxcem Elite cement. However, it is noteworthy that a higher mechanical property of the material does not necessarily translate to a more stable bond interface over time.

Although this study used single-rooted premolar with the same pattern, there are limiting factors that should be taken into consideration, such as the difficulty of performing the procedures especially in areas with difficult access and non-homogeneity of the substrate. Thus, future studies are required to complement discussions around the intracanal cementation process, such as the use of translucent posts.

## Conclusions

Based on the methodology performed, and the results of this study, it can be concluded that the silver nanoparticle could be used as a protocol for the use of this solution before glass fiber post cementation because when the silver nanoparticle solution was used, there was a tendency for the SUA and U200 resin cements to present higher values in the properties analyzed, with few alterations in the underlying dentin.
